# PMS1077 Sensitizes TNF-α Induced Apoptosis in Human Prostate Cancer Cells by Blocking NF-κB Signaling Pathway

**DOI:** 10.1371/journal.pone.0061132

**Published:** 2013-04-09

**Authors:** Jie Shi, Jing Chen, Nawal Serradji, Ximing Xu, Heng Zhou, Yinxing Ma, Zhihong Sun, Peng Jiang, Yuping Du, Jinbo Yang, Changzhi Dong, Qin Wang

**Affiliations:** 1 School of Life Sciences, Lanzhou University, Lanzhou, China; 2 Université Paris Diderot, Sorbonne Paris Cité, ITODYS, CNRS UMR 7086, Paris, France; 3 Université Paris Diderot, Sorbonne Paris Cité, Unité de Biologie Fonctionnelle et Adaptative, CNRS EAC4413, Paris, France; 4 Université Paris Diderot, Sorbonne Paris Cité, UMR S-698, Paris, France; Sun Yat-sen University Medical School, China

## Abstract

Our previous studies have demonstrated that PMS1077, a platelet-activating factor (PAF) antagonist, could induce apoptosis of Raji cells. However, the mechanism of action has not yet been determined. The nuclear transcription factor-kappa B (NF-κB) signaling pathway plays a critical role in tumor cell survival, proliferation, invasion, metastasis, and angiogenesis, so we determined the effects of PMS1077 and its structural analogs on tumor necrosis factor-α (TNF-α) induced activation of NF-κB signaling. In this study, we found that PMS1077 inhibited TNF-α induced expression of the NF-κB regulated reporter gene in a dose dependent manner. Western blot assay indicated that PMS1077 suppressed the TNF-α induced inhibitor of κB-α (IκB-α) phosphorylation, IκB-α degradation, and p65 phosphorylation. PMS1077 consistently blocked TNF-α induced p65 nuclear translocation as demonstrated in the immunofluorescence assay used. Docking studies by molecular modeling predicted that PMS1077 might interact directly with the IκB kinase-β (IKK-β) subunit. These results suggested that PMS1077 might suppress the activation of NF-κB by targeting IKK-β involved in the NF-κB signaling pathway. Finally, we showed that PMS1077 sensitized cells to TNF-α induced apoptosis by suppressing the expression of NF-κB regulated anti-apoptotic genes. Our results reveal a novel function of PMS1077 on the NF-κB signaling pathway and imply that PMS1077 can be considered as an anti-tumor lead compound.

## Introduction

In recent years, we have synthesized a certain number of piperazine derivatives which showed potent dual anti-PAF and anti-HIV-1 activities [1], [2], [3], [4]. While further improving these properties and taking into account the flexibility of these compounds, we were also engaged to study the potential effects of these compounds in other pathological conditions, such as inflammation and tumor genesis. Our previous work demonstrated that PMS1077 could induce apoptosis of Raji cells, but the mechanism of action remains unclear [5].

Because of the critical role of NF-κB regulated gene products in cellular proliferation, survival, invasion, metastasis, and angiogenesis [6], [7], [8], we reasoned that PMS1077 might mediate apoptosis of cancer cells by modulating the NF-κB signaling cascade. The NF-κB family is essentially composed of five proteins, including Rel-A (p65), Rel-B, C-Rel, p50, and p52 [9]. The typical mammalian NF-κB consists of a p50/p65 heterodimer. In unstimulated cells, NF-κB binds to inhibitor of κB proteins and is sequestered in the cytoplasm as an inactive complex. Upon stimulation for example by TNF-α, the signaling cascade leads to activation of the IκB kinase complex, which results in the phosphorylation, ubiquitination, and degradation of IκB by the 26S proteasome [9], [10]. Then, the liberated NF-κB heterodimer rapidly translocates into the nucleus, where it binds to the κB site and induces transcription of a wide variety of target genes involved in cancer development and progression [8], [11].

In the present study, we hypothesized that PMS1077 may modulate the NF-κB activation pathway. To test this hypothesis, we determined the effects of PMS1077 and its structural analogs on the TNF-α-induced NF-κB activation. Our findings showed that PMS1077 can inhibit the TNF-α induced IκB-α degradation, IκB-α phosphorylation, p65 phosphorylation, and p65 nuclear translocation. Docking studies by molecular modeling predicted that PMS1077 might suppress the NF-κB activation by directly interacting with IKK-β. In addition, PMS1077 was also found to suppress the TNF-α induced expression of NF-κB regulated anti-apoptotic genes, leading to sensitization of TNF-α induced apoptosis in tumor cells. In this way, data from this study advanced our understanding of the molecular mechanisms involved in the anticancer activity of PMS1077, and may help us to further optimize their properties for potential drug development.

## Materials and Methods

### Cell culture and reagents

HEK293T (human embryonic kidney), DU145 (human prostate cancer) and PC3 (human prostate cancer) cells were obtained from the American Type Culture Collection. Cells were cultured in DMEM (Dulbecco's modified Eagle's Medium) supplemented with 10% FBS (fetal bovine serum), 100 units/mL penicillin, and 100 mg/mL streptomycin and incubated at 37°C in a 5% CO_2_ incubator. PMS1077 and its structural analogs were synthesized as previously reported [4]. Those analogs were dissolved in DMSO to produce a 50 mmol/L stock solution for in vitro experiments. TPCA-1 (a specific inhibitor of NF-κB) was purchased from Sigma Aldrich (St. Louis, MO, U.S). Antibodies against P-p65, p65, P-IκB-α, IκB-α, Bcl-xL, Bcl-2, survivin and PARP were purchased from Cell Signaling Technology. TNF-α was purchased from Pepro Tech. GenEscort™ Transfection Reagent was purchased from Wisegen Biotechnology Corporation (Nanjing, China).

### Transfection and luciferase reporter assay

DU145 and PC3 cells were seeded into 60 mm tissue culture dishes, respectively. NF-κB -dependent firefly luciferase reporter plasmid (4×κB-pGL4.20) was transfected into seeded cells using GenEscort™ transfection reagent in accordance with the manufacturer's instructions. After 48 h, cells were selected and proliferated in medium supplemented with 5 µg/mL of puromycin until resistant clones appeared. The two validated clones were named DU145-NF-κB-Luc and PC3-NF-κB-Luc. High throughput screening for the indicated compounds was performed in these cell lines using ONE-Glo Luciferase Assay System (Promega).

To study the NF-κB activation in HEK293 cells by TNF-α, NF-κB-dependent firefly luciferase reporter (4×κB-pGL4.20) was transiently co-transfected along with the Renilla luciferase plasmid (pGL4.74) in HEK-293T cells line using a GenEscort™ transfection reagent in accordance with manufacturer's instructions. After treatment, cells were lysed with passive lysis buffer (Promega), and luciferase activity was determined using a Dual-Luciferase Reporter Assay System (Promega) with a Wallac1420 VICTOR (Perkin-Elmer, Wellesely, MA, U.S). Relative luciferase activity was expressed as a ratio of firefly luciferase activity to renilla luciferase activity. Data represent three independent experiments performed in triplicate.

### 3-(4, 5-cimethylthiazol-2-yl)-2, 5-diphenyl tetrazolium bromide (MTT) assay

Cell viability was determined using MTT assay. Briefly, cells were plated into 96-well plates (1×10^4^ cells/well), treated with various compounds at various concentrations, and then maintained in culture for a further 12 or 24 h. At the end of that period, 20 µL MTT (5 mg/mL) was added to the culture media and incubated at 37°C for 2 h. Then the medium was removed. The water-insoluble formazan crystals were then dissolved in DMSO (100 µL/well). The optical density of each well was measured with a Wallac1420 VICTOR (Perkin-Elmer, Wellesley, MA, U.S) at 570 nm with a reference wavelength of 630 nm. For each concentration tested, wells containing all reagents except for cells served as controls. Cell survival is here described as relative absorbance (A) percentage as defined by (A_drug_/A_control_×100) (Ye et al., 2004).

### Western blotting analysis

Western blotting of proteins was performed as described in one of our previous studies [12]. Briefly, treated cells were harvested in RIPA lysis buffer containing 50 mM Tris-HCl (pH 7.5), 150 mM NaCl, 1% NP-40, 0.5% DOC, 0.1% SDS, 5 mM EDTA, 1 mM EGTA, 20 mM NaF, 2 mM sodium orthovanadate and Protease Inhibitor Cocktail (Roche). After lysis, the lysates were centrifuged for 10 min at 13000 g at 4°C. Total protein samples (30–60 µg) were transferred onto PVDF membrane after electrophoretic separation in 12% SDS polyacrylamide gel. After blocking with 5% non-fat milk in TBST (0.1%) for 2 h at room temperature, the membranes were incubated overnight with the primary antibody at 4°C and washed five times in TBST, then incubated with the horseradish peroxidase conjugated secondary antibodies at room temperature for 2 h. The membranes were washed five times in TBST, and then incubated with an enhanced chemiluminescence western detection system (Perkin-Elmer Life Sciences, Boston, MA, U.S) and exposed to X-ray film.

### NF-κB/p65 nuclear translocation assay

Immunocytochemical analysis of NF-κB/p65 nuclear translocation in PC-3 cells was performed using a Cellular NF-κB Translocation Kit (Beyotime Biotech) according to the manufacturer's instructions, as previously described [13], [14]. Briefly, cells were seeded into 96-well plates at 4000 cells/well. 24 hours later, cells were pretreated with PMS1077 (50 µM) for 2 h, followed by treatment with 20 ng/ml TNF-α for 30 minutes. Cells pretreated with 0.2% DMSO and 2 µM TPCA-1 were used as negative and positive controls, respectively. After treatment, cells were fixed and incubated with a blocking buffer at room temperature for 1 h to suppress non-specific binding. Next, cells were incubated overnight with the primary NF-κB p65 antibody at 4°C, followed by incubation with a Cy3-conjugated secondary antibody at room temperature for 1 h, then with DAPI for 5 min before observation. The p65 protein appeared red under fluorescence microscopy and the nuclei appeared blue. The red and blue images were merged using Image J software to produce purple fluorescence in areas of co-localization.

Nuclear translocation of NF-κB/p65 was also detected in DU145 cells by Western blotting analysis of the presence of p65 in cytoplasm and nucleus. After treatment, the cells cytoplasmic and nuclear fractions were prepared using the method as described in one of our previously studies [12]. Then protein samples were analyzed by Western blotting.

### RT-PCR assay

Total RNA of the treated cells was prepared using the RNAprep pure cell Kit (TIANGEN, Bei Jing, China) according to the manufacturer's instructions. 5 µg of total RNA from each sample were subjected to reverse transcription using M-MLV reverse transcriptase (Promega). The following primers were used: GAPDH-forward, GTCAACGGATTTGGTCGTATT and GAPDH- reverse, AGTCTTCTGGGTGGCAGTGAT; Bcl-xL-forward, CTGAATCGGAGATGG AGACC and Bcl–xL-reverse, TGGGATGTCAGGTCACTGAA; Bcl-2-forward, TGCACCTGACGCCCT TCAC and AGACAGCCAGGAGAAATCAAACAG and Bcl-2-reverse, AGACAGCCAGGAGA AATCAAACAG; survivin-forward, CCTTTCCTAAGACATTGCTAAG and survivin-reverse, GTG AATTTTTGAAACTGGACAG. PCR was carried out at 94°C for 30 s, at 56°C for 30 s and 1 min at 70°C for 30 cycles. PCR products were analyzed on 2% agarose gel and visualized by ethidium bromide staining.

### Apoptosis assay

The detection of apoptotic cells was performed using FITC-conjugated Annexin-V and Propidium Iodide (PI). DU145 cells were treated with 0, 40 and 80 µM PMS-1077 for 24 h and then were then pelleted down by centrifugation, washed twice with cold PBS, and centrifuged at 1000 rpm to collect the cells. Cells were resuspended in 200 µL of binding buffer, and then 10 µL of Annexin-V-FITC, 5 µL of PI were added. Cells were incubated in dark for 30 min and analyzed by flow cytometry. FACS was performed using 488 nm as excitation wavelength and band pass filters of 515–545 nm (for Annexin-V-FITC detection) and 563–607 nm (for PI detection). Data analysis was performed using the Cell Quest program.

### Molecular docking study

The IKK-β kinase domain (residues 16–310) was extracted from crystal structure of IKK-β in complex with an inhibitor (PDB ID: 3RZF) [15]. Compounds PMS1077 and PMS601 were built using Avogadro, with MMFF94 force field energy minimization including 5000 steps of steepest descent and 2000 steps of conjugate gradients [16]. All the structures for docking were prepared using AutoDockTools [17]. Gasteiger charge and merging non-polar hydrogens were added.

Molecular docking was performed by Autodock Vina [18]. Compounds were set as flexible structures, and the binding conformers were searched using Iterated Local Search global optimizer. Final binding conformers were chosen according to the best AutoDock Vina score, combining of knowledge-based and empirical approach Eq. 1.
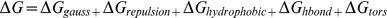
(1)


Here, *ΔG* is total binding energy; *ΔG_gauss_* is two gaussian functions containing attractive term of dispersion; *ΔG_repulsion_* is the square of *d* (surface distance) when *d<0*; *ΔG_hydrophobic_* and *ΔG_hbond_* are hydrophobic interactions and hydrogen bonds, respectively; and *ΔG_tors_* is compound rotatable bonds.

The binding affinity was also evaluated using AutoDock sore 4.1 (Eq. 2) and NNScore 2.0 [19].
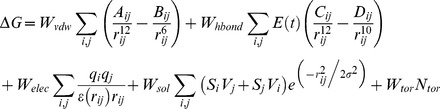
(2)


Here, *W_vdw_*, *W_hbond_*, *W_elec_*, and *W_sol_* are the weighting constants of Van der Waals interactions, hydrogen bonds, electrostatic interactions, and desolvation energy, respectively; *W_tor_* is the weight of compounds torsion.

NNScore 2.2 takes much more receptor-ligand characteristics to contribute for binding affinity, including AutoDock Vina's terms and 12 distinct binding characteristics from BINANA [20]. In the present study, BINANA and Ligplot+ [21] were introduced to analyze interaction of PMS1077 and IKK-β.

### Statistics and data analysis

All experiments were performed independently at least three times and the data are presented as mean±standard deviation unless otherwise indicated. Statistical significance between untreated groups and treated groups was determined using one-way analysis of variance (ANOVA) and the t-test. P<0.05 was considered significant.

## Results

### PMS1077 suppressed TNF-α induced expression of NF-κB-regulated reporter gene

**Figure 1:**
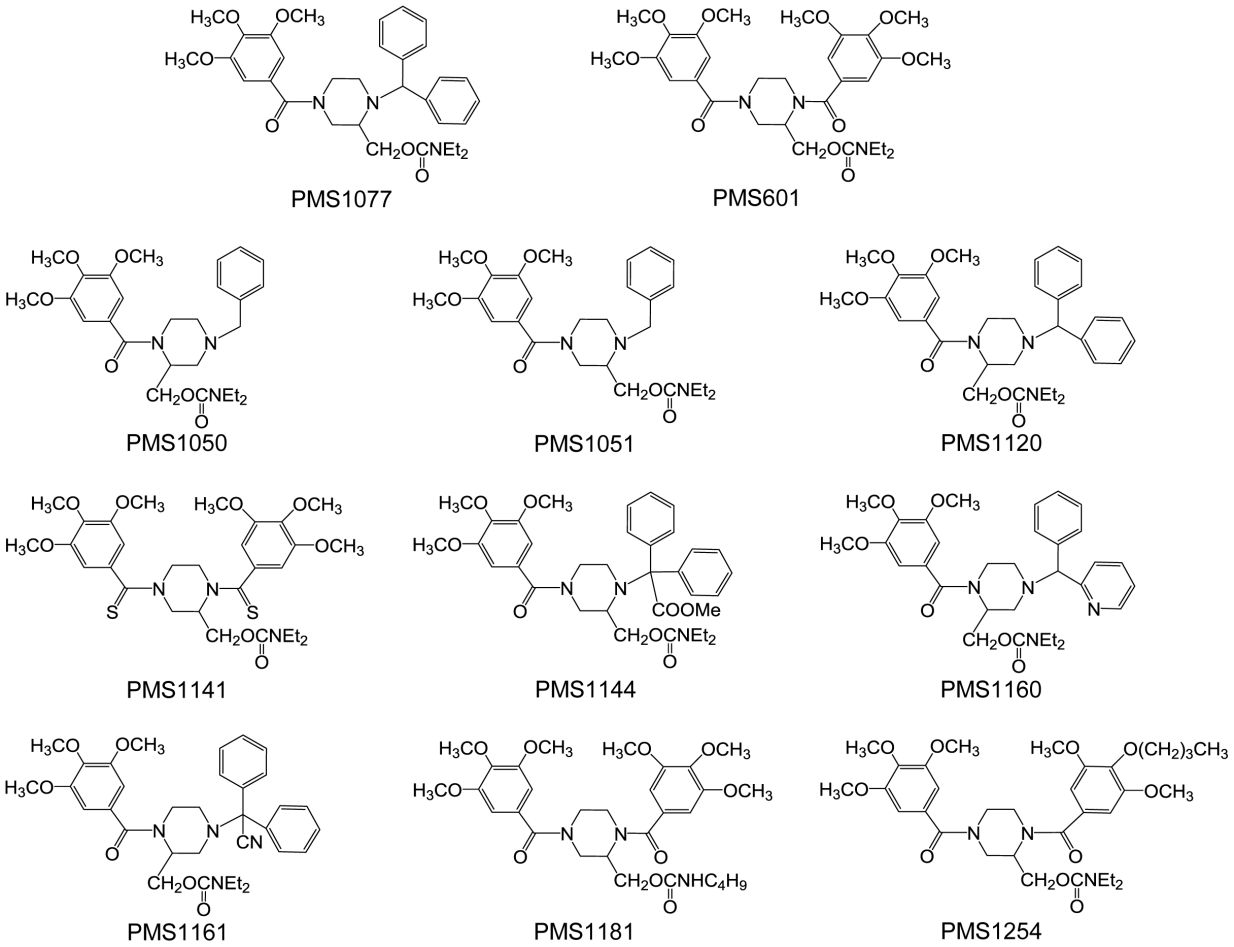
Structures of PMS1077 and its structural analogs.

**Figure 2:**
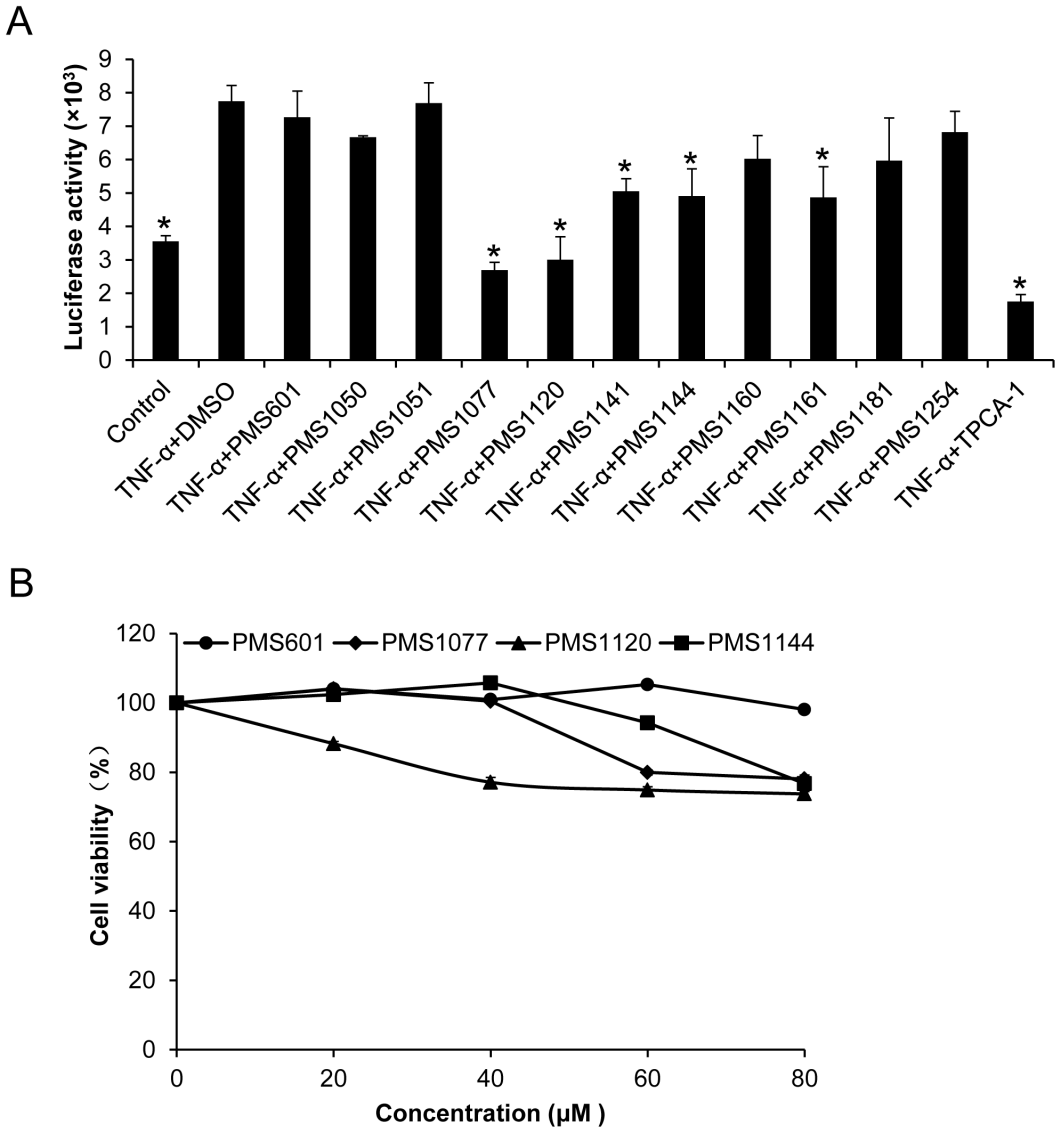
Effects of PMS1077 and its structural analogs on TNF-α induced NF-κB activation. (A) DU145-NF-κB-Luciferase cells were treated with PMS1077 and its structural analogs at final concentration of 50 µM. TPCA-1 (2 µM) is a specific inhibitor of NF-κB for positive control and DMSO as vehicle. Then cells were left untreated or exposed to TNF-α (20 ng/ml) for 12 h. Luciferase activity was measured using ONE-Glo® Luciferase Assay System (Promega). (B) DU145 cells were treated with PMS601, PMS1077, PMS1120 and PMS1144 at the indicated concentrations for 12 h. Cell viability was determined using MTT assay. The values are the mean±S.D. for three independent replicates. *P<0.05 versus TNF-α stimulated group.

In this study, to elucidate the mechanism involved in anti-tumor activities of PMS1077 (Fig. 1), we established DU145-NF-κB-Luc and PC3-NF-κB-Luc cell lines stably expressing a 4×κB promoter driving luciferase reporter and determined the effects of PMS1077 on the TNF-α induced activation of NF-κB in these cell lines. In this reporter gene assay, PMS1077 was observed to inhibit TNF-α induced NF-κB activity by more than 50% in DU145 cells and PC3 cells. Only the data in DU145 cells are shown in Fig. 2A.

In order to find more potent compounds, a series of analogs (Fig. 1), structurally very close to PMS1077, were submitted to the same assay. PMS1120, a position isomer of PMS1077 showed the comparative activities as PMS1077 in both cell lines, while PMS1141 was as active as PMS1077 only in PC3 cell line. Other analogs were less active than PMS1077 in both the cell lines and PMS601 was one of the most inactive compounds in this series. As for PMS1077, only the data in DU145 cells are shown in Fig. 2A.

To exclude the effects of cytotoxicity, we determined the effects of these compounds on cell viability. MTT assay indicated that PMS601, PMS1077, PMS1120 and PMS1141 only showed moderate cytotoxicity (<30%) in DU145 cells at concentrations as high as 80 µM (Fig. 2B). Finally, we selected PMS1077 as the best compound and PMS601 as control for the subsequent experiments.

**Figure 3:**
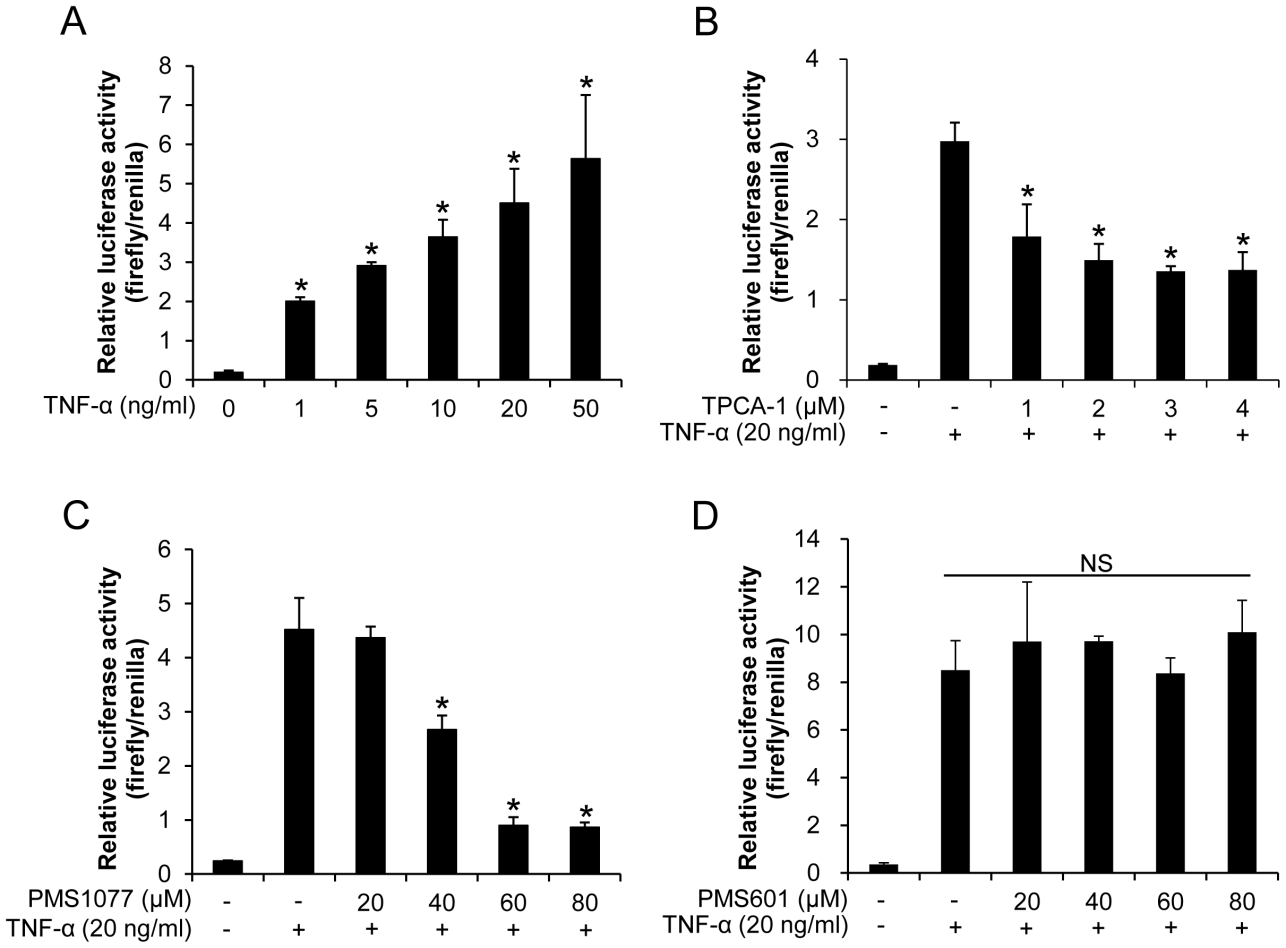
PMS1077 inhibited TNF-α induced expression of NF-κB regulated reporter gene. HEK293T cells were transiently co-transfected with NF-κB-Firefly luciferase and TK-Renilla Luciferase reporter vectors for NF-κB activity assay. (A) NF-κB-dependent reporter gene expression induced by TNF-α. Transfected cells were treated with vehicle or TNF-α (1, 5, 10, 20 and 50 ng/ml) for 12 h; (B) TPCA-1 inhibited the expression of the TNF-α induced NF-κB dependent reporter gene. Transfected cells were treated with vehicle or TPCA-1(1, 2, 3, and 4 µM), then incubated with TNFα (20 ng/ml) for 12 h; (C) PMS1077 inhibited TNF-α induced, NF-κB dependent reporter gene expression. Transfected cells were treated with vehicle or PMS1077 (20, 40, 60 and 80 µM), then incubated with TNF-α (20 ng/ml) for 12 h; (D) PMS601 showed no effect on TNF-α induced NF-κB dependent reporter gene expression. Transfected cells were treated with vehicle or PMS601 (20, 40, 60 and 80 µM), then incubated with TNF-α (20 ng/ml) for 12 h. After treatment, luciferase activity in (A), (B), (C), and (D) were measured using a dual luciferase assay system (Promega). Data shown represent the relative luciferase activity normalized against Renilla luciferase activity. The values are the mean±S.D. for three independent replicates. *P<0.05 versus TNF-α stimulated group; NS, not significant.

To further validate these results in the more accurate dual-luciferase assay system, HEK293T cells were transiently co-transfected with NF-κB-regulated luciferase reporter plasmid (4×κB-minip/PGL4.20) and internal control plasmid (PGL4.74). As shown in Fig. 3A, NF-κB relative luciferase activity was induced by TNF-α in a dose dependent manner, and this activity was suppressed by a known IKK-β inhibitor TPCA-1(Fig. 3B). When cells were treated with PMS1077, we found that TNF-α induced NF-κB relative luciferase reporter gene activity was suppressed in a dose dependent manner (Fig. 3C). However, PMS601, a negative control, showed no significant effect on this activity (Fig. 3D).

### PMS1077 inhibited TNF-α dependent IκB-α phosphorylation and degradation

**Figure 4:**
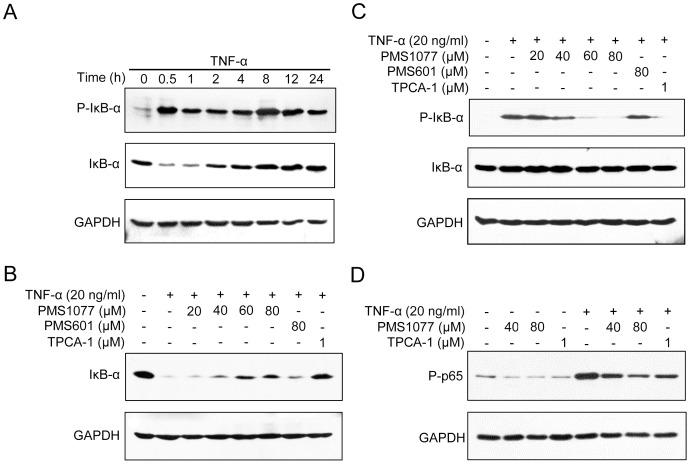
PMS1077 inhibited TNF-α induced IκB-α degradation, IκB-α phosphorylation, p65 phosphorylation. (A) TNF-α induced IκB-α degradation, IκB-α phosphorylation. HEK293T cells were treated with TNF-α (20 ng/ml) for the indicated periods of time; (B) PMS1077 inhibited TNF-α induced IκB-α degradation. HEK293T cells were treated with vehicle or PMS1077 (20, 40, 60 and 80 µM) or PMS601 (80 µM) and TPCA-1(1 µM), then incubated with TNF-α (20 ng/ml) for 0.5 h; (C) PMS1077 inhibited TNF-α induced IκB-α phosphorylation. HEK293T cells were treated with vehicle or PMS1077 or PMS601 or TPCA-1 as in (B), then incubated with TNF-α (20 ng/ml) for 2 h; (D) DU145 cells were treated with vehicle or PMS1077 (40 and 80 µM) and TPCA-1(1 µM), then cells were either left untreated or exposed to TNF-α (20 ng/ml) for 12 h. After treatment, the whole cell lysates in (A), (B), (C), and (D) were prepared and analyzed by Western blotting with antibodies for IκB-α, phosphorylation-IκB-α (Ser-32), phosphorylation-p65 (Ser-536) and GAPDH. All data shown are representative of three independent experiments.

The phosphorylation and ubiquitin proteasome-mediated degradation of IκB-α protein plays a critical role in the activation of NF-κB signaling pathway. While detecting phosphorylation and degradation of IκB-α in HEK293T, we found that TNF-α induced IκB-α degradation reached the maximum at 0.5 h to 1 h and that resynthesis of IκB-α occurred 2 h to 4 h after TNF-α treatment (Fig. 4A). To determine whether inhibition of TNF-α induced NF-κB activation is caused by suppression of IκB-α degradation, we pretreated HEK293T cells with PMS1077 and then exposed them to TNF-α for 0.5 h. Notably, PMS1077 was found to inhibit degradation of IκB-α in a dose dependent manner (Fig. 4B). In contrast, we observed no obvious effect on degradation of IκB-α in PMS601 (80 µM) pretreated cells (Fig. 4B). These data indicated that PMS1077 can suppress TNF-α induced IκB-α degradation, which leads to suppression of NF-κB activation.

To determine whether inhibition of TNF-α induced degradation of IκB-α was caused by inhibition of phosphorylation of IκB-α, we studied the effects of PMS1077 on TNF-α induced phosphorylation of IκB-α in HEK293T cells. As shown in Fig. 4A, resynthesis of IκB-α reached a level similar to that of the control 2–4 h after treatment and IκB-α phosphorylation reached a moderate level at 1–2 h after TNF-α treatment, so we performed western blot analysis of IκB-α phosphorylation 2 h after TNF-α stimulation. The results of this analysis showed that pretreatment of PMS1077 strongly inhibited TNF-α induced IκB-α phosphorylation in a dose dependent manner, but PMS601 showed no effect on this phosphorylation (Fig. 4C).

Taken together, these results indicated that PMS1077 inhibited TNF-α induced activation of NF-κB via suppressing phosphorylation and degradation of IκB-α. However, the structural analog PMS601 showed no significant effect on phosphorylation and degradation of IκB-α.

### PMS1077 inhibited constitutive and TNF-α induced phosphorylation of p65

It has been well demonstrated that the phosphorylation of serine 536 of p65 plays an important role in the regulation of NF-κB activity and can be induced by a variety of pro-inflammatory stimuli, including TNF-α [22], [23], [24], [25]. For this reason, we also investigated the effects of PMS1077 on TNF-α induced phosphorylation of p65. Western blot analysis showed that phosphorylation of p65 occurred in TNF-α treated DU145 cells and this phosphorylation of p65 was inhibited in a dose dependent manner in the pretreated cells with PMS1077 (40 and 80 µM) (Fig. 4D, right).

DU145 and PC3 cells are known to have constitutively active NF-κB [26], [27]. To determine whether PMS1077 also affects the constitutive NF-κB expression in these cells, we treated DU145cells with PMS1077 (40 and 80 µM) without TNF-α. As shown in Figure. 4D (left), PMS1077 completely suppressed the constitutive phosphorylation of p65 in DU145 cells in a dose dependent manner. In addition, PMS601 was found to have no significant effect on both the inducible and constitutive phosphorylation of p65 (Fig. S1). Thus, these data indicated that PMS1077 can suppress both the inducible and constitutive NF-κB activation.

### PMS1077 blocked TNF-α induced NF-κB/p65 nuclear translocation

**Figure 5:**
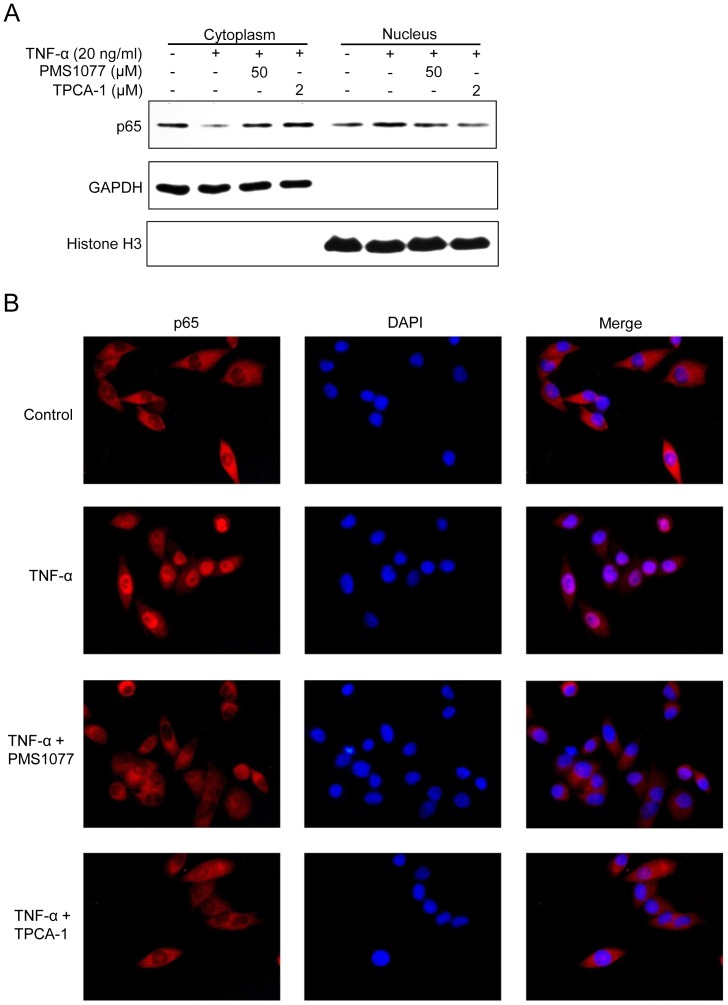
PMS1077 inhibited TNF-α-induced NF-κB/P65 nuclear translocation. (A) DU145 cells were pretreated with PMS1077 (50 µM) or TPCA-1(2 µM) for 6 h and followed by TNF-α (20 ng/ml) stimulation for 0.5 h. Cytoplasmic and nuclear extracts representing equal numbers of cells were analyzed by Western blotting using the indicated antibodies. (B) PC-3 cells were pretreated with PMS1077 (50 µM) for 6 h and followed by TNF-α (20 ng/ml) stimulation for 0.5 h. TPCA-1 (2 µM) and DMSO were used as positive NF-κB inhibitor and negative control, respectively. After treatment, cells were stained with primary anti-p65 antibody and Cy3 fluorescein-conjugated secondary antibody (Red), and then the nucleus was counterstained with DAPI (blue) and examined using Fluorescence microscopy. Images were acquired for each fluorescence channel, using suitable filters with 40×objective. The red and blue images were merged using Image J software. All data shown are representative of three independent experiments.

Because IκB-α degradation is required for nuclear translocation of p65, we sought to determine whether PMS1077 could also suppress the TNF-α induced nuclear translocation of p65. Western blot analysis showed that nuclear translocation of p65 occurred in DU145 after TNF-α treatment. However, when the cells were pretreated with PMS1077 (50 µM) or TPCA-1, TNF-α failed to induce nuclear translocation of p65 (Fig. 5A). To confirm these results, we performed immunocytochemistry to observe p65 in PC3 and DU145 cells. The results indicated that in untreated, as well as PMS1077 (50 µM) or TPCA-1 pretreated cells, p65 was localized in the cytoplasm, whereas in cells treated with TNF-α alone, p65 was translocated to the nucleus (Fig. 5B and Fig.S2). However, PMS601 showed no effect on this translocation of p65 (Fig. S2). These data confirmed that PMS1077 blocked the TNF-α induced nuclear translocation of p65.

### Docking study by molecular modeling of interactions between PMS1077 or PMS601 and IKK-β

**Figure 6:**
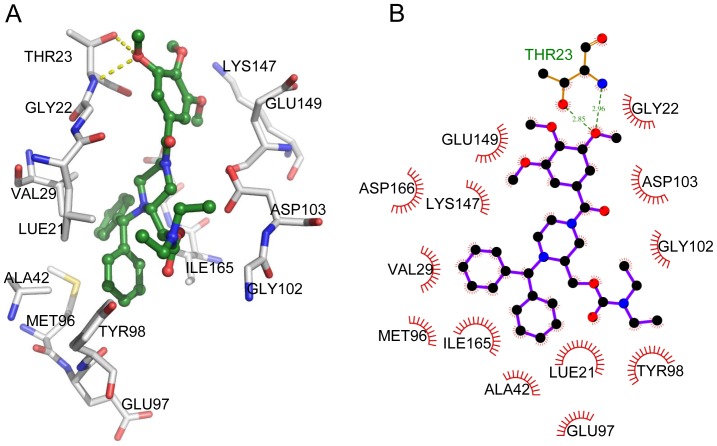
The model of PMS1077 binding to IKK-β. (A) The 3D model of PMS1077 (ball and stick style) interaction with IKK-β kinase domain (residues shown in stick model), hydrogen bonds are shown in dashed line. (B) The 2D diagram of interactions between PMS1077 and IKK-β.

A variety of agents, including TNF-α, interleukin-1 beta (beta), phorbol myristate acetate (PMA), and okadaic acid (OA) have been reported to activate NF-κB, and PMA can induce activation of NF-κB through the IKK complex [28], [29]. In the present study, PMS1077 can both inhibit TNF-α and PMA induced NF-κB activity (Fig. 2A and Fig. S3). The TNF-α induced phosphorylation of IκB-α is catalyzed by IKK. Based on the fact that PMS1077 can inhibit IκB-α phosphorylation, but PMS601 showed no effects on this phosphorylation (Fig. 4C), we speculated whether PMS1077 could target IKK. In order to explore this possibility, we compared the binding affinity of these two compounds and IKK-β using computational methods. PMS1077 and PMS601 were docked to the IKK-β kinase domain, and binding affinity was estimated using three different score functions which were AutoDock vina score, AutoDock score 4.1 and NNScore 2.0 (see Materials and methods). From AutoDock vina score, the score of PMS1077 is lower than that PMS601, and the calculated K_d_ of PMS1077 was approximatively 15 times lower than that of PMS601. This was confirmed by other two scoring system (Table 1). Both AutoDock score 4.1 and NNScore 2.0 showed that the calculated K_d_ of PMS1077 was more than two hundreds lower than that of PMS601. This calculation is in agreement with the results given above (Fig. 4B), which showed no obvious change in the phosphorylation of IκB-α at PMS601 concentrations of up to 80 µM. The details of the interactions between PMS1077 and IKK-β were shown in Fig. 6. Hydrophobic interactions were found to contribute most in the binding affinity, such as residues LUE21, VAL29, TYR98, and ILE165. Two hydrogen bonds formed between THR23 and PMS1077.

**Table 1:** Comparison binding affinity between compounds and IKK-β by 3 different scores.

	Vina score		Autodock score 4.1		NNScore 2.0	
Compound	Score (kcal/mol)	K_d_ (µM)	Score (kcal/mol)	K_d_ (µM)	Average score*	K_d_ (µM)
PMS1077	−8.30	0.82	−7.94	1.38	8.19±1.99	6.45
PMS601	−6.70	12.27	−4.66	385.18	5.83±1.84	1470.00

* The higher the better.

In this way, these calculations revealed that PMS1077 might inhibit NF-κB activation by directly binding to IKK-β and different binding affinity to IKK-β resulting in different activity of PMS1077 and PMS601.

### PMS1077 sensitized prostate cancer cells to TNF-α induced apoptosis through suppressing TNF-α induced transcription of anti-apoptotic genes

**Figure 7:**
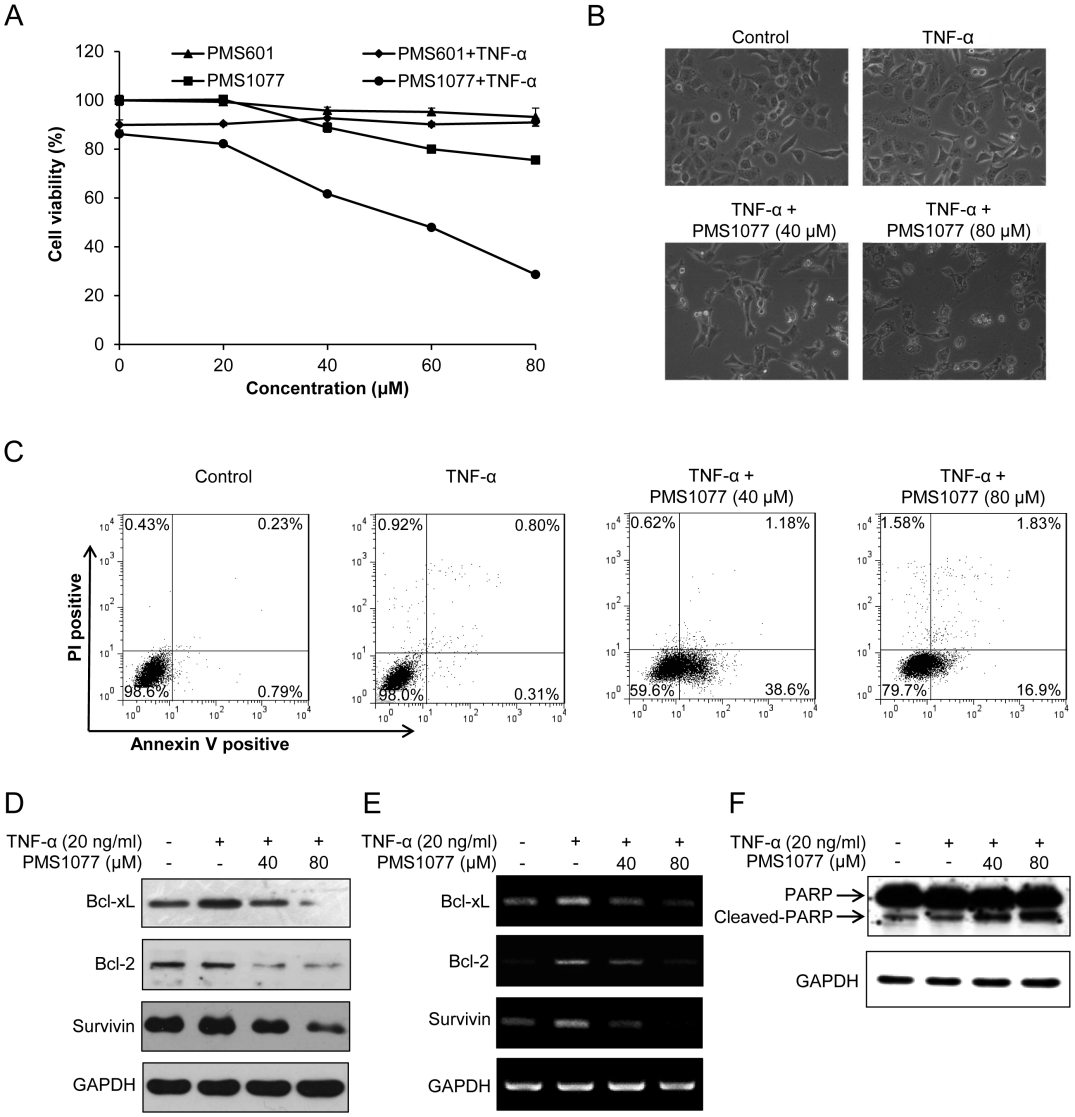
PMS1077 sensitized cancer cells to TNF-α induced apoptosis and inhibited the TNF-α induced expression of anti-apoptotic gene. (A) DU145 cells were treated with vehicle, PMS1077 or PMS601 as the indicated concentration (0–80 µM) and then incubated with or without TNF-α (20 ng/ml) for 24 h. Cell viability was determined by MTT assay. (B) DU145 cells were treated with vehicle or PMS1077 (40 and 80 µM), and then incubated with TNF-α (20 ng/ml) for 24 h. Treated cells were observed by differential interference contrast microscope. (C) DU145 cells were treated as (B) and apoptosis analysis was performed by flow cytometry through annexin-V-FITC and Propidium iodide (PI) staining treated cells. (D) DU145 cells were treated with vehicle or PMS1077 (40 and 80 µM), then incubated with TNF-α (20 ng/ml) for 12 h. After treatment, the whole cell lysates were prepared and analyzed by Western blotting with antibodies as indicated. (E) DU145 cells were incubated with TNF-α (20 ng/ml) for 1 h, with vehicle or PMS1077 pretreatment for 6 h. The mRNA level of NF- κB target genes were examined using RT–PCR. (F) DU145 cells were treated as (D) and whole cell lysates were prepared and analyzed by Western blotting with antibodies for PARP and GAPDH. All data shown are representative of three independent experiments.

Our previous work has demonstrated that PMS1077 could induce apoptosis of Raji cells [5]. In this work, we found that PMS1077 also induced apoptosis in DU145 and significantly sensitized TNF-α induced apoptosis, as evidenced by MTT assay (Fig. 7A) and by differential interference contrast microscopy (Fig. 7B). This was further confirmed by flow cytometry through annexin-V-FITC and Propidium iodide (PI) staining of the PMS1077 treated DU145 cells as shown in Fig. 7C.

It has been well established that inhibition of NF-κB signaling pathway sensitizes TNF-α induced cell death and TNF-α is often used to provoke cell apoptosis [30], [31], [32]. NF-κB signaling antagonizes TNF-α and chemotherapeutic agents induced apoptosis by promoting the transcription of anti-apoptotic genes, such as c-FLIP, cIAP-1, cIAP-2, XIAP, survivin, Bcl-xL and Bcl-2. In this way, blocking of NF-κB signaling can potentiate TNF-α induced apoptosis [31], [32], [33], [34]. We then determined whether PMS1077 can modulate the TNF-α induced expression of the anti-apoptosis genes Bcl-xL, Bcl-2 and survivin in DU145 cells. Notably, after TNF-α treatment, the expression of these genes was enhanced, but pretreatment of the cells with PMS1077 down-regulated the expression of these genes in a dose dependent manner both at protein and mRNA level (Fig. 7D and 7E). We also found that TNF-α induced cleavage of PARP was significantly increased by PMS1077 (Fig. 7F). Taken together, these results indicated that PMS1077 can sensitize DU145 cells to TNF-α induced apoptosis.

## Discussion

In this study, we investigated the mechanism by which PMS1077 mediates antitumor effects. PMS compounds are tri-substituted derivatives of piperazine and are originally synthesized for their anti-PAF and anti-HIV-1 properties. The most studied compound, PMS601, has been demonstrated to be capable of crossing the blood-brain-barrier and considered to be a promising drug candidate for HIV-associated dementia (HAD) therapy [1], [2], [3]. While improving the known properties of these compounds, our interests were also extended to the eventual effects of these compounds to other pathologies, such as inflammation and tumor genesis. Our previous work revealed that PMS1077 can induce apoptosis of Raji cells, but its mechanisms of action has not been addressed [5].

In order to understand the anti-tumor effect of PMS1077, our attention was focused on the NF-κB signaling pathway. NF-κB has been linked to tumor survival, proliferation, invasion, metastasis, angiogenesis, and chemoresistance [7], [8], [35]. The present study showed that PMS1077 inhibited TNF-α induced phosphorylation and degradation of IκB-α and TNF-α induced translocation of p65 to the nucleus. Molecule docking studies predicted that PMS1077 might interact directly with IκB kinase-β (IKK-β) subunit. The collective findings suggested that PMS1077 might suppress NF-κB activation by directly binding to IKK-β.

NF-κB signaling antagonizes TNF-α and chemotherapeutic agents induced apoptosis by inducing the transcription of anti-apoptotic genes, such as c-FLIP, cIAP-1, cIAP-2, XIAP, survivin, Bcl-xL, and Bcl-2 [34], [35]. TNF-α can trigger both NF-κB and apoptosis signaling simultaneously, so suppression TNF-α induced NF-κB signaling can potentiate TNF-α induced apoptosis [31], [32], [33], [34], [36], [37]. In the present work, we found that PMS1077 can down-regulate the TNF-α induced expression of the anti-apoptotic Bcl-xL, Bcl-2 and survivin gene, and significantly potentiates TNF-α induced cleavage of PARP. These results indicate that PMS1077 has a sensitization effect on TNF-α induced apoptosis through suppressing the NF-κB regulated anti-apoptotic gene expression induced by TNF-α.

In conclusion, our results indicate that PMS1077 sensitizes cells to TNF-α induced apoptosis through suppressing the TNF-α induced activation of NF-κB signaling and the subsequent expression of NF-κB regulated anti-apoptotic gene. Accordingly, the present data partially explain the mechanism underlying the anti-tumor activities of PMS1077. In future studies, more work will be performed to identify the molecular target of this compound and optimize its bioactive properties.

Taken together, data from this study reveal a novel function of PMS1077 on NF-κB activity and suggest that PMS1077 can be considered as an anticancer lead compound.

## Supporting Information

Figure S1 PMS601 showed no effects on both constitutive and TNF-α induced phosphorylation of p65. DU145 cells were treated with vehicle or PMS601 (80 µM), then cells were either left untreated or exposed to TNF-α (20 ng/ml) for 12 h. The whole cell lysates were prepared and analyzed by Western blotting with antibodies for phosphorylation-p65 (Ser-536) and GAPDH. All data shown are representative of three independent experiments.
(TIF)

Figure S2 PMS1077 rather than PMS601 inhibited TNF-α-induced NF-κB/P65 nuclear translocation. DU145 cells were pretreated with PMS1077 (50 µM) or PMS601 (50 µM) for 6 h and followed by TNF-α (20 ng/ml) stimulation for 0.5 h. TPCA-1 (2 µM) and DMSO were used as positive NF-κB inhibitor and negative control, respectively. After treatment, cells were stained with primary anti-p65 antibody and Cy3 fluorescein-conjugated secondary antibody (Red), and then the nucleus was counterstained with DAPI (blue) and examined using Fluorescence microscopy. Images were acquired for each fluorescence channel, using suitable filters with 40×objective. The red and blue images were merged using Image J software. All data shown are representative of three independent experiments.
(TIF)

Figure S3 PMS1077 inhibited PMA induced expression of NF-κB regulated reporter gene. PC3-NF-κB-Luciferase cells were treated with PMS1077 or PMS601 at final concentration as indicated. TPCA-1 (2 µM) is a specific inhibitor of NF-κB for positive control and DMSO as vehicle. Then cells were left untreated or exposed to PMA (20 ng/ml) for 12 h. Luciferase activity was measured using ONE-Glo® Luciferase Assay System (Promega).
(TIF)
