# Supplementary figures and images for: PMS1077 Sensitizes TNF-α Induced Apoptosis in Human Prostate Cancer Cells by Blocking NF-κB Signaling Pathway
Source: PLoS One. 2013 Apr 9;8(4):e61132. doi: 10.1371/journal.pone.0061132 (PMC3621893; doi:10.1371/journal.pone.0061132)

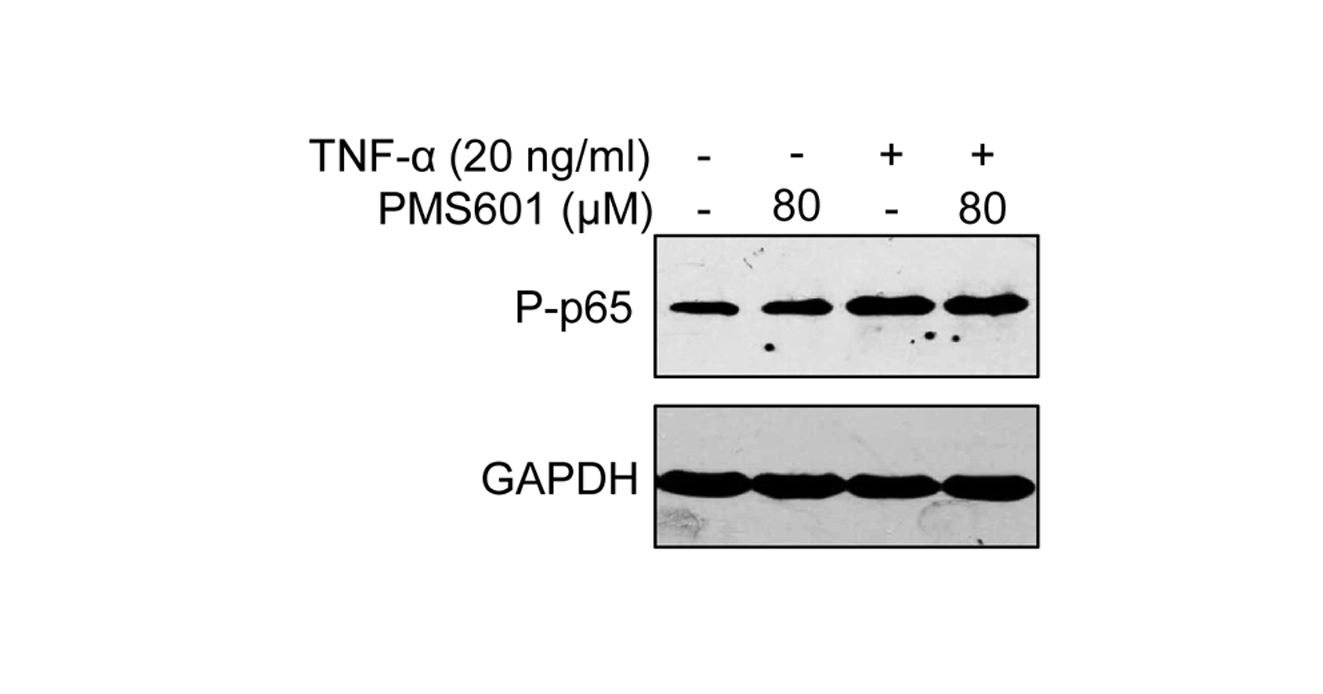

Supplement: Figure S1 — PMS601 showed no effects on both constitutive and TNF-α induced phosphorylation of p65. DU145 cells were treated with vehicle or PMS601 (80 µM), then cells were either left untreated or exposed to TNF-α (20 ng/ml) for 12 h. The whole cell lysates were prepared and analyzed by Western blotting with antibodies for phosphorylation-p65 (Ser-536) and GAPDH. All data shown are representative of three independent experiments. (TIF) [file pone.0061132.s001.tif]

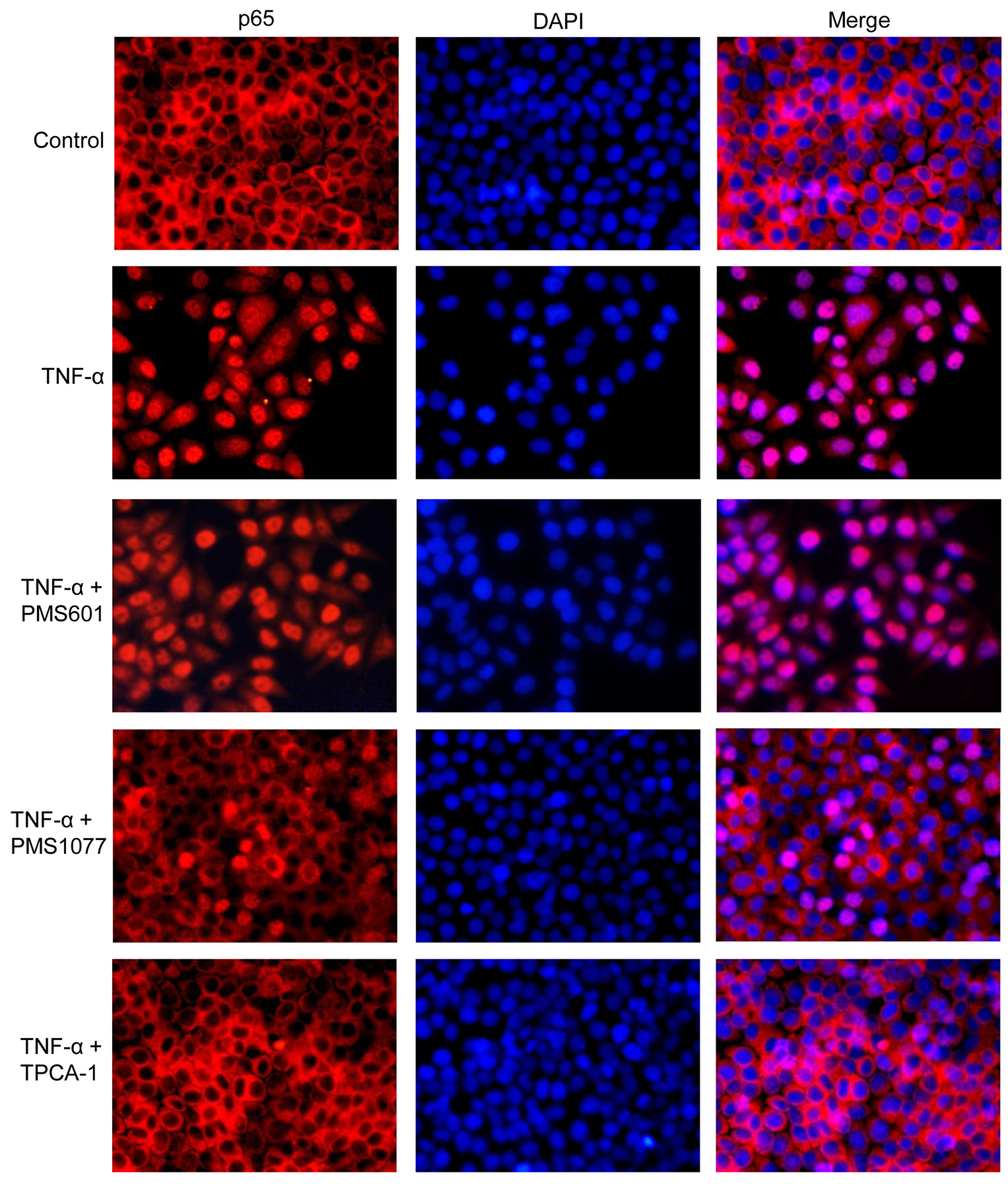

Supplement: Figure S2 — PMS1077 rather than PMS601 inhibited TNF-α-induced NF-κB/P65 nuclear translocation. DU145 cells were pretreated with PMS1077 (50 µM) or PMS601 (50 µM) for 6 h and followed by TNF-α (20 ng/ml) stimulation for 0.5 h. TPCA-1 (2 µM) and DMSO were used as positive NF-κB inhibitor and negative control, respectively. After treatment, cells were stained with primary anti-p65 antibody and Cy3 fluorescein-conjugated secondary antibody (Red), and then the nucleus was counterstained with DAPI (blue) and examined using Fluorescence microscopy. Images were acquired for each fluorescence channel, using suitable filters with 40×objective. The red and blue images were merged using Image J software. All data shown are representative of three independent experiments. (TIF) [file pone.0061132.s002.tif]

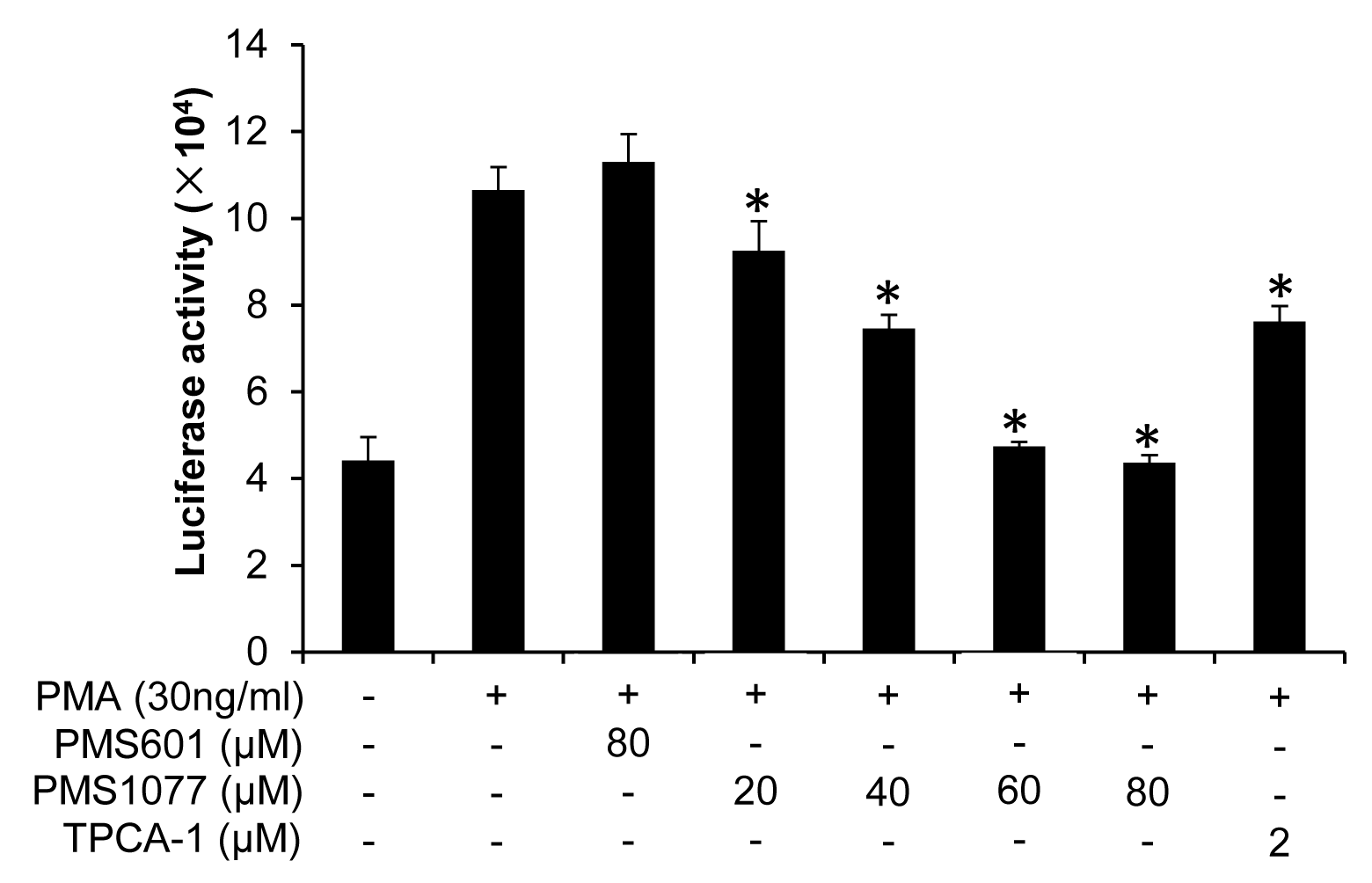

Supplement: Figure S3 — PMS1077 inhibited PMA induced expression of NF-κB regulated reporter gene. PC3-NF-κB-Luciferase cells were treated with PMS1077 or PMS601 at final concentration as indicated. TPCA-1 (2 µM) is a specific inhibitor of NF-κB for positive control and DMSO as vehicle. Then cells were left untreated or exposed to PMA (20 ng/ml) for 12 h. Luciferase activity was measured using ONE-Glo® Luciferase Assay System (Promega). (TIF) [file pone.0061132.s003.tif]
